# Supplementary material for: A single main-chain hydrogen bond required to keep GABAA receptors closed
Source: Nat Commun. 2025 Jul 3;16:6107. doi: 10.1038/s41467-025-61447-0 (PMC12222489; doi:10.1038/s41467-025-61447-0)
Supplement: Supplementary file 1 — Supplementary Information [file 41467_2025_61447_MOESM1_ESM.pdf]

A single main-chain hydrogen bond required to keep $GABA_A$ receptors closed

Cecilia M. Borghese[1], Jason D. Galpin[2], Samuel Eriksson Lidbrink[3], Yuxuan Zhuang[3], Netrang G. Desai[1], Rebecca J. Howard[3,4], Erik Lindahl[3,4], Christopher A. Ahern[2], and Marcel P. Goldschen-Ohm[1]*

[1]Department of Neuroscience, University of Texas at Austin, Austin, TX, USA
[2]Department of Molecular Physiology and Biophysics, University of Iowa, Iowa City, IA, USA
[3]Department of Biochemistry and Biophysics, Stockholm University, Stockholm, Sweden
[4]Department of Applied Physics, KTH Royal Institute of Technology, Stockholm, Sweden

Supplementary Figure/Table	Page
Supplementary Table 1	2
Supplementary Table 2	3
Supplementary Table 3	4
Supplementary Figure 1	5-6
Supplementary Figure 2	7
Supplementary Figure 3	8
Supplementary Figure 4	9
Supplementary Figure 5	10
Supplementary Figure 6	11-12
Supplementary Figure 7	13

Supplementary Table 1 | Summary of results from Figs. 2-3, expressed as median (interquartile range)

Receptor	I_{total} (μA)	*Unliganded P_O*	ΔG (kcal · mol^{-1})	n*
α1(L9'T) β2 γ2	3.7 (2.8−4.2)	0.20 (0.12−0.37)	0.82 (0.33−1.21)	5
α1(L9'T, Val279Val) β2 γ2	1.61 (0.92−2.78)	0.12 (0.10−0.12)	1.18 (1.18−1.30)	7
α1(L9'T, Val279Vah) β2 γ2	2.86 (0.90−14.9)	0.19 (0.18−0.21)	0.86 (0.78−0.90)	3
α1(L9'T, Val279Blank) β2 γ2	0.17 (0.11−0.29)	-	-	8
α1(L9'T) β2 γ2	0.98 (0.61−1.31)	0.24 (0.18−0.35)	0.68 (0.36−0.92)	8
α1(L9'T) β2(Ile275Ile) γ2	1.22 (0.55−1.83)	0.19 (0.14−0.28)	0.90 (0.55−1.13)	16
α1(L9'T) β2(Ile275Iah) γ2	0.37 (0.15−0.83)	0.81 (0.75−0.92)	-0.86 (-1.41 − -0.64)	17
α1(L9'T) β2(Ile275Blank) γ2	0.025 (0.017−0.037)	-	-	15
α1 β2 γ2	7.6 (4.9−10.9)	0.0015 (0.0000−0.0038)	3.6 (3.0−4.0)	12
α1 β2(Ile275Ile) γ2	1.46 (0.86−2.31)	0.0050 (0.0005−0.018)	2.5 (2.4−3.3)	9
α1 β2(Ile275Iah) γ2	0.49 (0.25−0.67)	0.071 (0.058−0.102)	1.5 (1.3−1.6)	8
α1 β2(Ile275Blank) γ2	0.012 (0.000−0.032)	-	-	8

ΔG is the closed versus open free energy difference for unliganded receptors (Eq. 1) and n is the number of oocytes.

*In a few cases, the unliganded open probability (P_o) was indistinguishable from zero, and the corresponding ΔG could not be calculated.

Supplementary Table 2 | Parameters for Hill equation fits to GABA and diazepam concentration-response data

Receptor	GABA EC_{50} (μM)	n_H	I_{max}	n
α1(L9'T) β2 γ2	0.78 (0.66 to 0.94)	0.87 ± 0.05	0.99 ± 0.01	5
α1(L9'T, Val279Val) β2 γ2	1.73 (1.14 to 2.82)	0.80 ± 0.10	0.98 ± 0.04	7
α1(L9'T, Val279Vah) β2 γ2	1.04 (0.78 to 1.39)	0.80 ± 0.06	0.98 ± 0.02	6
α1(L9'T) β2 γ2	0.46 (0.39 to 0.55)	0.79 ± 0.04	0.98 ± 0.01	5
α1(L9'T) β2(Ile275Ile) γ2	2.56 (1.66 to 4.33)	0.56 ± 0.05	1.04 ± 0.03	5
α1(L9'T) β2(Ile275Iah) γ2	1.68 (1.39 to 2.07)	0.53 ± 0.02	1.03 ± 0.01	8
α1 β2 γ2	101 (83 to 126)	0.99 ± 0.07	1.00 ± 0.02	8
α1 β2(Ile275Ile) γ2	157 (137 to 181)	0.93 ± 0.04	1.02 ± 0.01	4
α1 β2(Ile275Iah) γ2	2.90 (2.33 to 3.67)	0.80 ± 0.05	1.02 ± 0.02	6

Receptor	Diazepam EC_{50} (μM)	n_H	I_{max}	n
α1(L9'T) β2 γ2	0.072 (0.040 to 0.270)	0.92 ± 0.17	0.99 ± 0.05	5
α1(L9'T, Val279Val) β2 γ2	0.074 (0.030 to 6.63)	0.95 ± 0.28	0.99 ± 0.08	4
α1(L9'T, Val279Vah) β2 γ2	0.100 (0.036 to 1710)	0.94 ± 0.31	1.01 ± 0.11	4

Hill equation (Eq. 2) parameters are from fits to the normalized responses. EC_{50} is expressed as mean (95% coefficient interval), n_H and I_{max} are expressed as mean ± SEM, and n is the number of oocytes.

Supplementary Table 3 | Molecular dynamics simulation parameters

	BIC*	GABA (desensitized)*	GABA +DZ*	GABA +ETO*	GABA +PPF*	GABA (open)**
Simulation box	127Å × 127Å × 163Å	127Å × 127Å × 163Å	127Å × 127Å × 163Å	127Å × 127Å × 163Å	127Å × 127Å × 163Å	140Å × 140Å × 160Å
Number of atoms	259,364	255,698	254,400	254,757	262,546	269,056
Number of waters	58,264	57,066	57,254	56,729	59,324	61,003
Salt concentration	150mM NaCl	150mM NaCl	150mM NaCl	150mM NaCl	150mM NaCl	150mM NaCl
Number of lipids	424 POPC	424 POPC	424 POPC	424 POPC	424 POPC	211 cholesterol, 130 POPC, 141 POPE, 42 POPS, 16 PtdIns(4,5)P2

*Simulations available at https://doi.org/10.5281/zenodo.8142630
**Simulations available at https://doi.org/10.5281/zenodo.10964268
POPC, 1-palmitoyl-2-oleoyl-*sn*-glycero-3-phosphocholine; *POPE*, 1-palmitoyl-2-oleoyl-*sn*-glycero-3-phosphoethanolamine; *POPS*, 1-palmitoyl-2-oleoyl-*sn*-glycero-3-phospho-l-serine; *PtdIns(4,5)P2*, phosphatidylinositol 4,5-bisphosphate.

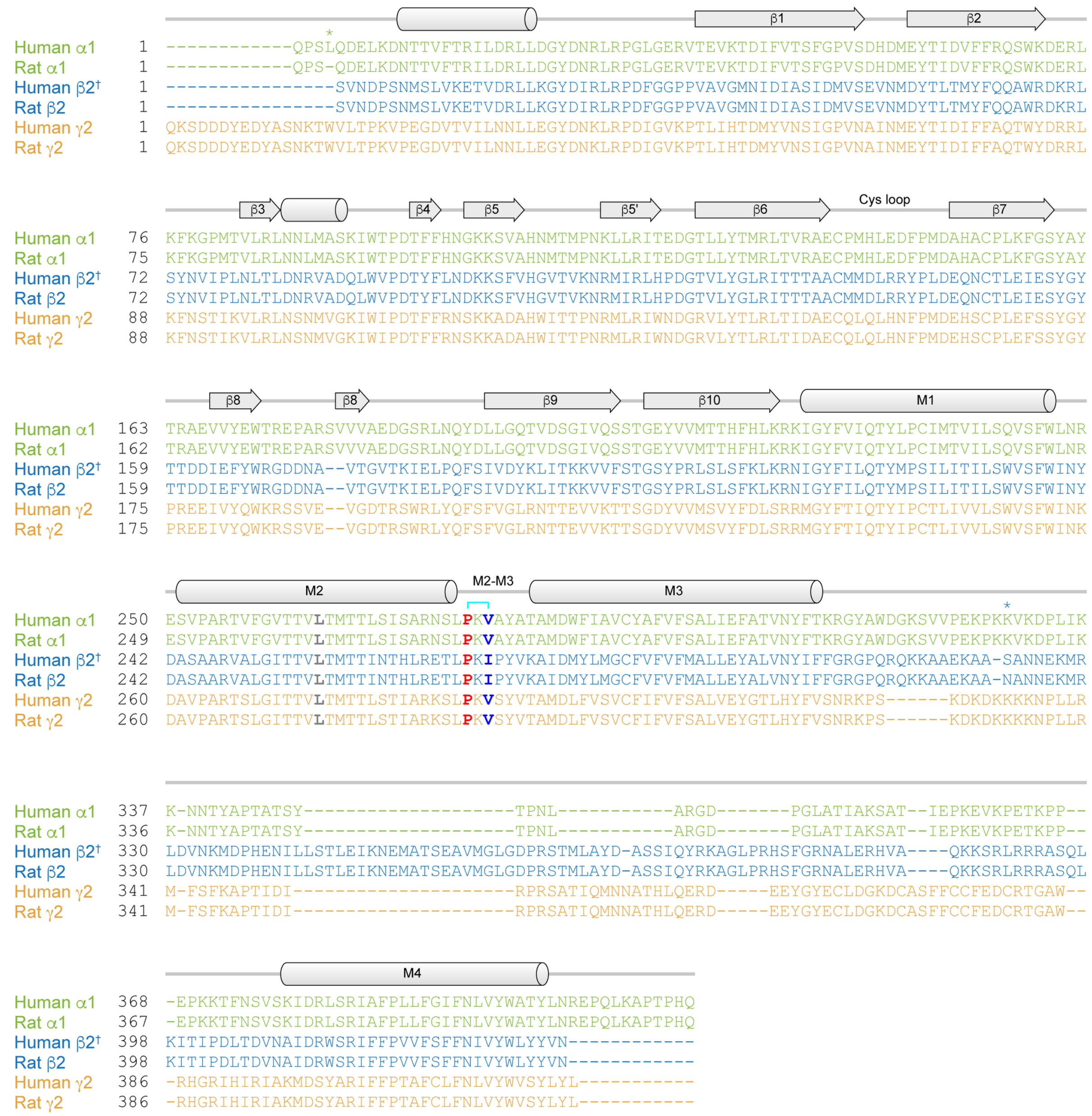

Supplementary Figure 1 | Human and rat sequences of $GABA_A$ subunits. Human and rat sequences are either identical (γ2) or differ by either a single residue gap in the N-terminus (α1) or a single residue substitution in the M3-M4 linker (β2). Residue numbering is for the mature protein. Notably, human and rat residue numbering is shifted by one for most of the α1 subunit. Approximate locations of helix and sheet secondary structures are indicated above the sequences. The main-chain H-bond donor (blue) and acceptor (red) residues in the M2-M3 linkers and the

main pore gate 9′ leucine (gray) are bolded. Protein sequence identifiers from UniProt are P14867 (human α1), P62813 (rat α1), P47870-1 (human β2, †short isoform), P63138 (rat β2), P18507 (human γ2), and Q6PW52 (rat γ2). The canonical β2 subunit (P47870-2, long isoform) includes an additional stretch of 38 amino acids in the intracellular M3-M4 linker compared to the short isoform.

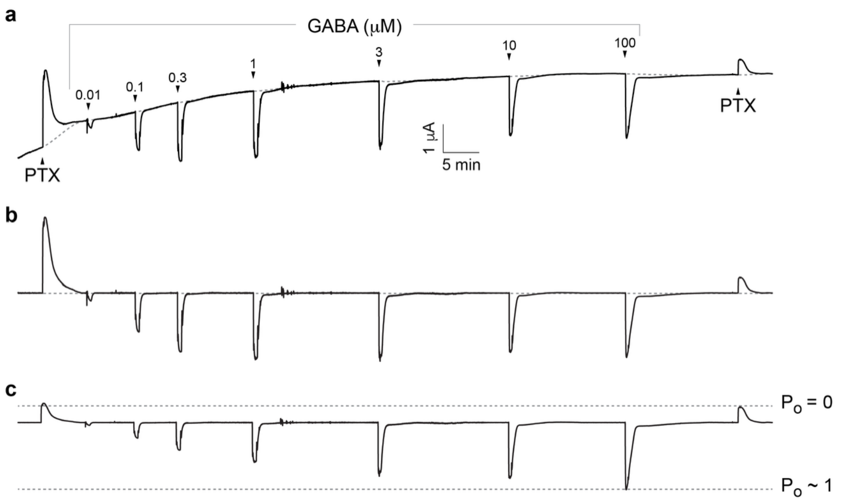

Supplementary Figure 2 | Detrending rundown of current time series traces as described in methods. a, Current trace for α1(Leu9′Thr,Val279Vah)β2γ2 receptors illustrating pulse protocol. Arrows indicate approximate onset of pulses of either 1 mM PTX or increasing concentrations of GABA. Dashed gray line is a spline fit to the basal current level between ligand applications. Rundown of constitutive PTX-sensitive current amplitude interpreted as a time-dependent loss of active channels. **b**, Baselined current after subtraction of the spline approximation to the baseline constitutive current from the raw current time series in panel a. **c**, Detrended current after dividing the baselined current in panel b by the magnitude of the spline approximation to the constitutive current (i.e., a value proportional to the number of active channels) and finally rescaling to match the original amplitude of the final response to PTX. This procedure reasonably accounts for the observed rundown in the number of active channels as evidenced by the similar PTX-sensitive current amplitudes at the beginning and end of the detrended trace despite not enforcing this a priori. Most importantly, this detrending has almost no effect on the relative magnitude of the final responses to saturating GABA and PTX at the end of the recording from which our primary conclusions are drawn.

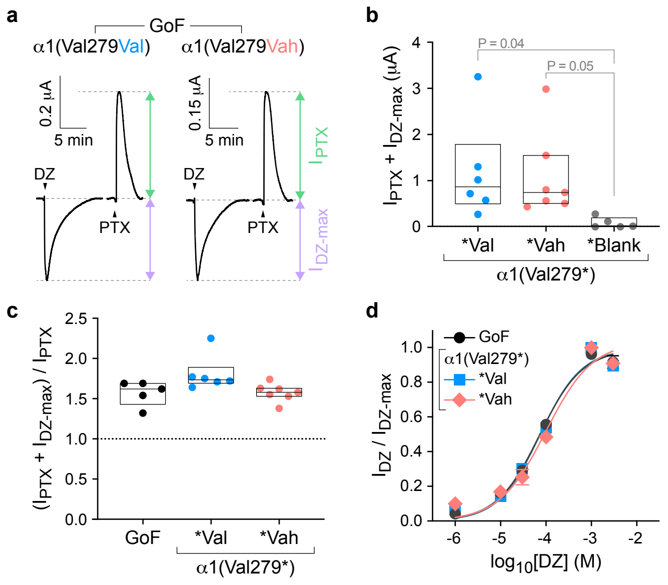

Supplementary Figure 3 | H-bond in α1 does not affect diazepam (DZ) modulation. a, Current responses to 10-20 s pulses (arrows) of DZ eliciting a maximal response or 1 mM PTX for α1(Leu9′Thr)β2γ2 (GoF) receptors after nonsense suppression incorporation of either the wild-type amino acid (Val) or its cognate α-hydroxy acid (Vah) at α1(Val279*). Vertical bars indicate the relative magnitude of PTX-sensitive (green) to DZ-elicited (purple) currents. **b**, Total current per oocyte as illustrated in panel a suggests reliable nonsense suppression incorporation of AA and ncAA with little to no read-through (i.e., relative lack of current for *blank). The notation Val279* denotes a TAG stop codon at position 279, and *Val or *Vah imply nonsense suppression incorporation of Val or Vah at the TAG site resulting in Val279Val or Val279Vah, respectively. *Blank indicates an unchanged TAG stop codon. Box plots show median and interquartile intervals. P-values ≤ 0.05 for Brown-Forsythe ANOVA with posthoc Dunnett's T3 test shown (left-to-right: n= 6,7,5). **c**, Maximal fold-potentiation of basal unliganded current by DZ (left-to-right: n= 5,6,7). **d**, Normalized concentration-response relations for DZ-elicited currents. Data are mean ± SEM across oocytes [n per condition: GoF, 5; α1(Val279Vah), 4; α1(Val279Val), 4].

Curves are the Hill equation fit to the means (**Eq. 2**). See **Supplementary Table 2** for fit parameters.

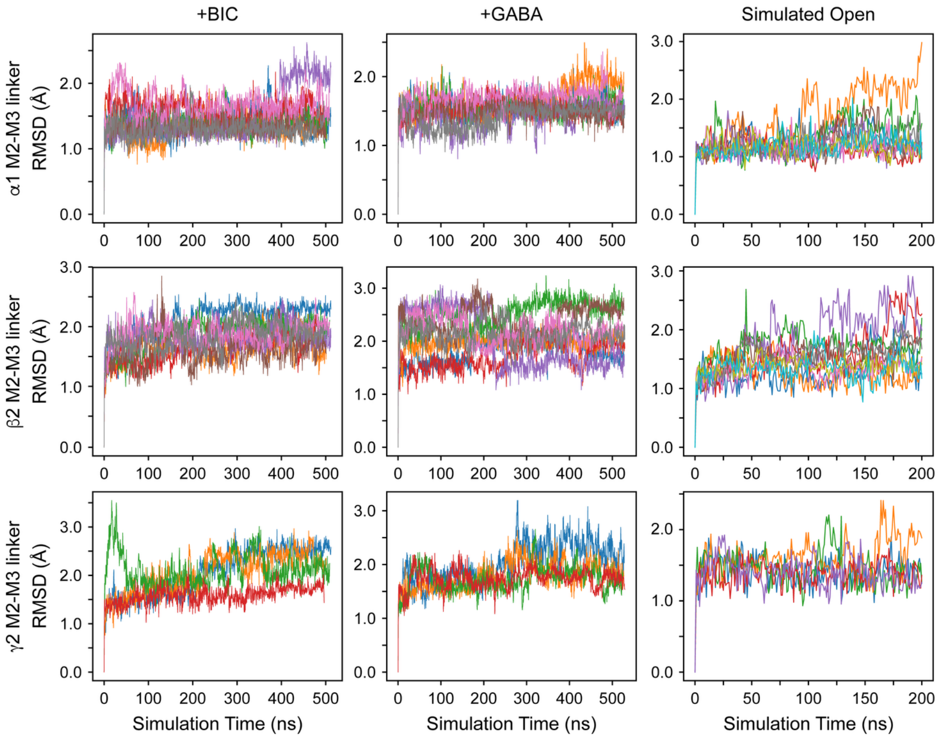

Supplementary Figure 4 | Convergence of MD simulations for M2-M3 linkers in each subunit.

Left panels: antagonist-bound complexes (+BIC, bicuculine; PDB 6X3S). Middle panels: GABA-bound complexes (+GABA; PDB 6X3Z). Right panels: simulated open state [43]. Distinct chains for each of four (+BIC, +GABA) or five (simulated open) replicates are colored differently.

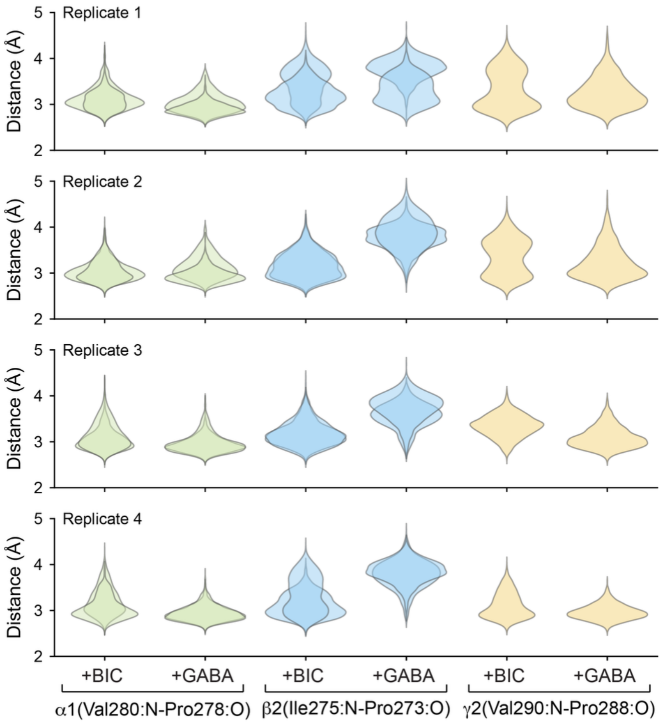

Supplementary Figure 5 | Chain-specific distance distributions for pairs of donor/acceptor atoms predicted to form a main-chain H-bond within the M2-M3 linker from MD simulations of antagonist- and GABA-bound complexes. Results shown are for either antagonist-bound (+BIC, bicuculine; PDB 6X3S) or GABA-bound (+GABA; PDB 6X3Z) complexes. The distributions are shown per replicate, with overlapping distributions corresponding to the two distinct chains for α1 and β2 subunits within the receptor.

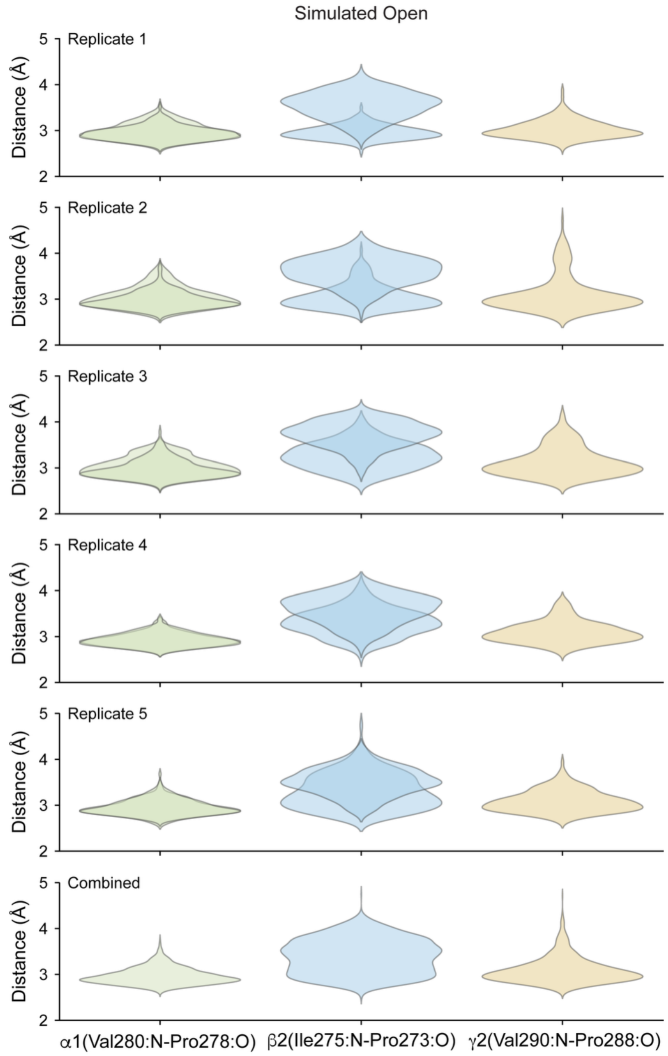

Supplementary Figure 6 | Chain-specific distance distributions for pairs of donor/acceptor atoms forming putative main-chain H-bonds within the M2-M3 linker from MD simulations of a simulated open conformation. Results shown are for a simulated open state conformation [43]. The distributions are shown per replicate, with overlapping distributions corresponding to the two distinct chains for α1 and β2 subunits within the receptor. The bottom panel shows the combined distributions across all replicates. Interestingly, a longer H-bond distance as compared to the closed structure was primarily evident in one of the two β2 subunits in each replicate, possibly reflecting the more pronounced asymmetry of the open versus desensitized states, and/or of the computational model versus experimental structures. Nonetheless, this simulated open

model supports our central identification of a state-dependent change in a β2-subunit main-chain H-bond.

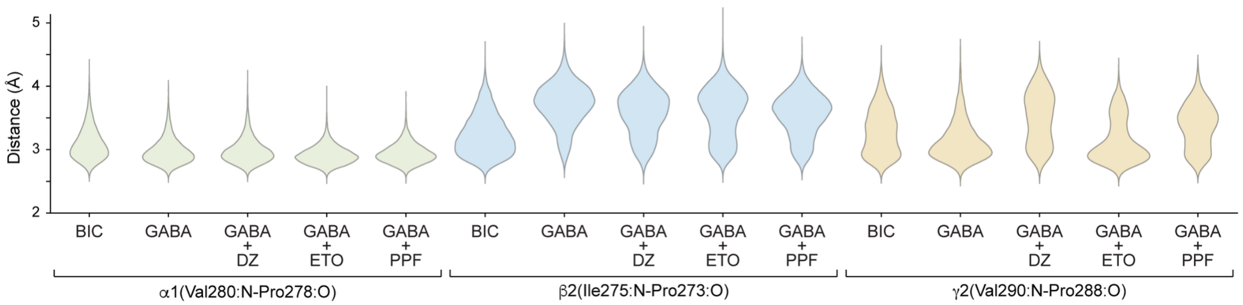

Supplementary Figure 7 | Subunit-specific distance distributions for pairs of donor/acceptor atoms forming putative main-chain H-bonds within the M2-M3 linker from MD simulations of various ligand complexes. Results shown are for MD simulations initiated from cryo-EM structures with antagonist-bound (BIC, bicuculine; PDB 6X3S), GABA-bound (PDB 6X3Z), or GABA in complex with the positive allosteric modulators diazepam (DZ; PDB 6X3X), etomidate (ETO; PDB 6X3V), or propofol (PPF; PDB 6X3T). The distributions are the combined distributions across all chains and replicates for each subunit.
