# Supplementary material for: A single main-chain hydrogen bond required to keep GABAA receptors closed
Source: Nat Commun. 2025 Jul 3;16:6107. doi: 10.1038/s41467-025-61447-0 (PMC12222489; doi:10.1038/s41467-025-61447-0)
Supplement: Supplementary file 2 — Description of Additional Supplementary Files [file 41467_2025_61447_MOESM2_ESM.pdf]

Description of Additional Supplementary Files

Supplementary Data 1. pUNIV-rat Gabra1. Rat Gabra1 sequence encoding the $GABA_A$ a1 subunit in pUNIV vector (Accession # NM_183326.2).

Supplementary Data 2. pUNIV-rat Gabra1(L263T). Rat Gabra1 sequence encoding $GABA_A$ a1(Leu9'Thr) subunit subcloned in pUNIV vector.

Supplementary Data 3. pUNIV-rat Gabra1(L263T-V279tag). Rat Gabra1 sequence encoding $GABA_A$ a1(Leu9'Thr,Val279tag) subunit subcloned in pUNIV vector.

Supplementary Data 4. pUNIV-rat Gabrb2. Rat Gabrb2 sequence encoding the $GABA_A$ b2 subunit subcloned in pUNIV vector (Accession # NM_012957.3).

Supplementary Data 5. pUNIV-rat Gabrb2(I275tag). Rat Gabrb2 sequence encoding the $GABA_A$ b2(Ile275tag) subunit subcloned in pUNIV vector.

Supplementary Data 6. pUNIV-rat Gabrg2. Rat Gabrg2 sequence encoding the $GABA_A$ g2 subunit subcloned in pUNIV vector (Accession # AY574252.1).
