# Supplementary material for: A single main-chain hydrogen bond required to keep GABAA receptors closed
Source: Nat Commun. 2025 Jul 3;16:6107. doi: 10.1038/s41467-025-61447-0 (PMC12222489; doi:10.1038/s41467-025-61447-0)
Supplement: Supplementary file 4 — Supplementary Data 2 [file 41467_2025_61447_MOESM4_ESM.pdf]

Sequence: pUNIV-rat Gabra1(L263T).dna (Circular / 7051 bp)
Enzymes: Unique 6+ Cutters (43 of 666 total)
Features: 16 total

Unique Cutters **Bold**

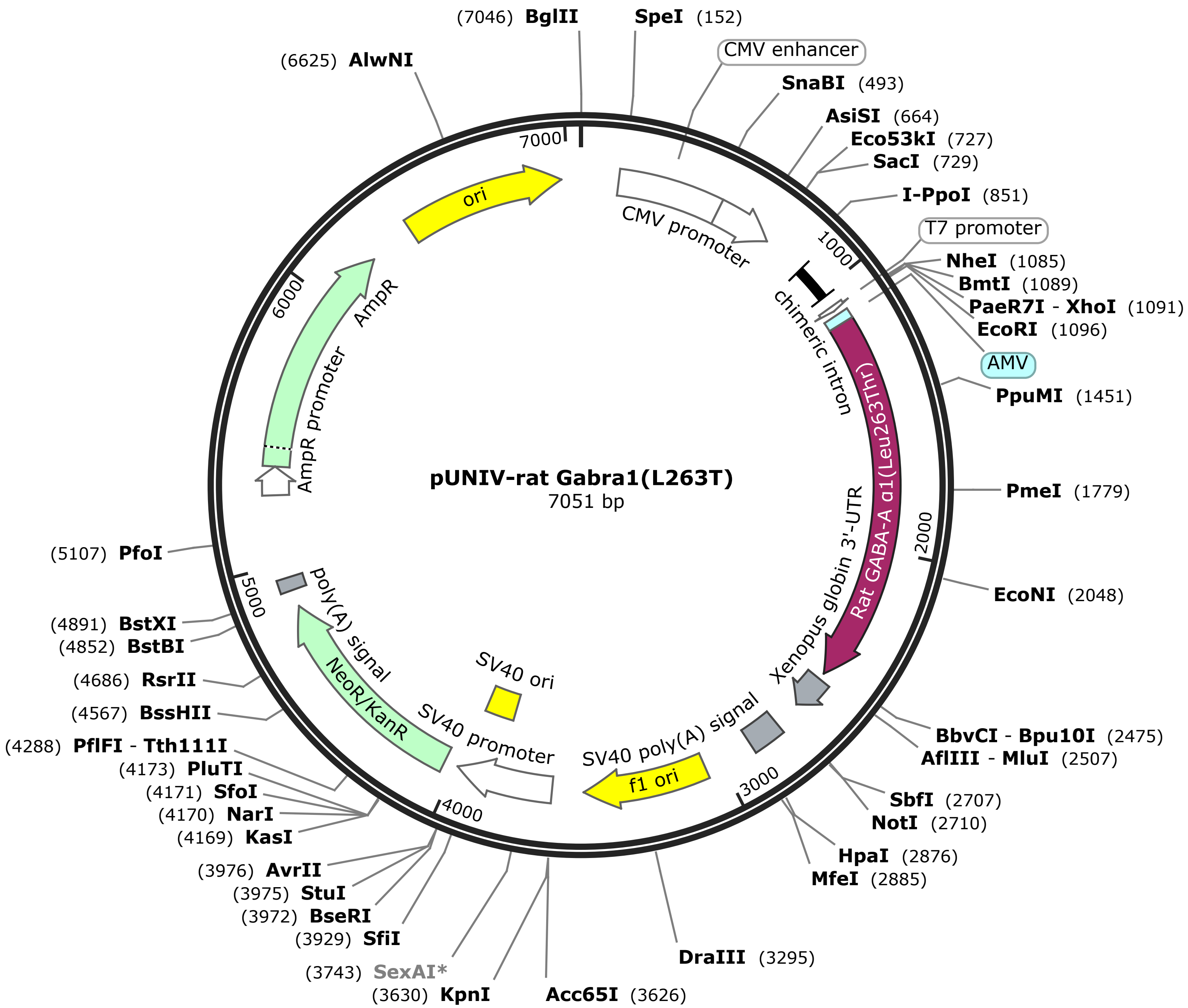

```
•••
tcaatattggccattagccatattattcattggttatatagcataaatcaatatt 55
ggctattggccattgcatacgttgtatctatatcataatatgtacatttatattg 110
gctcatgtccaatatgaccgccatgttggcattgattattgactagttattaata 165
gtaatcaattacggggtcattagttcatagcccatatatggagttccgcgttaca 220
taacttacggtaaatggcccgcctggctgaccgcccaacgacccccgcccattga 275
cgtcaataatgacgtatgttcccatagtaacgccaatagggactttccattgacg 330
tcaatgggtggagtatttacggtaaactgcccacttggcagtacatcaagtgtat 385
catatgccaagtccgccccctattgacgtcaatgacggtaaatggcccgcctggc 440
attatgcccagtacatgaccttacgggactttcctacttggcagtacatctacgt 495
attagtcatcgctattaccatggtgatgcggttttggcagtacaccaatgggcgt 550
ggatagcggtttgactcacggggatttccaagtctccaccccattgacgtcaatg 605
ggagtttgttttggcaccaaaatcaacgggactttccaaaatgtcgtaacaactg 660
cgatcgcccgccccgttgacgcaaatgggcggtaggcgtgtacggtgggaggtct 715
atataagcagagctcgtttagtgaaccgtcagatcactagaagctttattgcggt 770
agtttatcacagttaaattgctaacgcagtcagtgcttctgacacaacagtctcg 825
aacttaagctgcagtgactctcttaaggtagccttgcagaagttggtcgtgaggc 880
actgggcaggtaagtatcaaggttacaagacaggtttaaggagaccaatagaaac 935
tgggcttgtcgagacagagaagactcttgcgtttctgataggcacctattggtct 990
tactgacatccactttgcctttctctccacaggtgtccactcccagttcaattac 1045
agctcttaaggctagagtacttaatacgactcactataggctagcctcgagaatt 1100
ccgttttattttaatttctttcaaatacttccaccatgaagaaaagtcgggg 1155
tctctctgactatctttgggcctggaccctcattctgagcactctctcgggaaga 1210
agctatggacagccctcccaagatgaacttaaggacaacaccactgtcttcacga 1265
ggattttggaccgactgctggatggttatgacaatcgtctgagaccaggcttggg 1320
agagcgtgtaactgaagtgaagacggacatctttgtcaccagtttcggacccgtg 1375
tcagaccacgatatggaatatacaatagatgtgtttttccgccaaagctggaagg 1430
atgaaagattaaaattcaaaggacccatgacagtgctccggctgaacaacctgat 1485
ggccagtaaaatctggactccagatacatttttccacaatggaaaaaagtctgtg 1540
gcccacaacatgaccatgcccaataaactcctgcgtatcacagaggatggcacac 1595
tgctgtacaccatgaggttgactgtgagagccgaatgccccatgcacttagaaga 1650
ctttcccatggatgcccatgcctgcccactaaaatttgggagctatgcttataca 1705
agagcagaagttgtctatgagtggacaagggagccagcccgctcagtggttgtgg 1760
cagaagatgggtcacgtttaaaccagtatgaccttcttgggcaaacagttgactc 1815
tggaattgttcagtccagtactggagaatatgtggttatgacgactcactttcac 1870
ttgaagagaaaaatcggctactttgttattcaaacatatctgccatgcataatga 1925
cagtcattctctcccaagtctccttctggcttaacagagagtcagtaccagcaag 1980
aactgtctttggagtgacgaccgttaccaccatgacaaccttgagtatcagtgcc 2035
agaaattccctcccaaaggtggcttatgcaacggccatggactggtttattgcag 2090
tgtgctatgccttcgtgttctcggctctgattgagtttgccacagtaaactattt 2145
caccaagagagggtatgcgtgggatggcaaaagcgtggttccagaaaagccaaag 2200
aaagtgaaggatcctctcattaagaaaaacaacacatatgctcctacagcaacca 2255
gctataccccctaacttagccaggggtgaccccggcttggcaactattgctaaaag 2310
tgcgaccatagaaccgaaagaagtcaagcctgagacaaaaccgccagaacccaag 2365
aaaacctttaacagcgtcagcaaaatcgaccgactgtcaagaatagcctttccgc 2420
tgctatttggaatctttaacttagtctattgggccacgtatttaaacagagagcc 2475
tcagctaaaagcccccacaccccatcaatgaacgcgtgatctggttaccactaaa 2530
ccagcctcaagaacacccgaatggagtctctaagctacataataccaacttacac 2585
tttacaaaatgttgtccccccaaaatgtagccattcgtatctgctcctaataaaaa 2640
gaaagtttcttcacattctaaaaaaaaaaaaaaaaaaaaaaaaaaaaaaaaaccc 2695
cccccccctgcaggcggccgcttccctttagtgagggttaatgcttcgagcagac 2750
atgataagatacattgatgagtttggacaaaccacaactagaatgcagtgaaaaa 2805
aatgctttatttgtgaaatttgtgatgctattgctttatttgtaaccattataag 2860
ctgcaataaacaagttaacaacaacaattgcattcattttatgtttcaggttcag 2915
ggggagatgtgggaggttttttaaagcaagtaaaacctctacaaatgtggtaaaa 2970
tccgataaggatcgatccgggctggcgtaatagcgaagaggcccgcaccgatcgc 3025
ccttcccaacagttgcgcagcctgaatggcgaatggacgcgccctgtagcggcgc 3080
attaagcgcggcgggtgtggtggttacgcgcagcgtgaccgctacacttgccagc 3135
gccctagcgcccgctcctttcgctttcttcccttcctttctcgccacgttcgccg 3190
gctttccccgtcaagctctaaatcgggggctccctttagggttccgatttagtgc 3245
tttacggcacctcgaccccaaaaaacttgattagggtgatggttcacgtagtggg 3300
```

```
ccatcgccctgatagacggtttttcgccctttgacgttggagtccacgttcttta 3355
atagtggactcttgttccaaactggaacaacactcaaccctatctcggtctattc 3410
ttttgatttataagggattttgccgatttcggcctattggttaaaaaatgagctg 3465
atttaacaaaaatttaacgcgaattttaacaaaatattaacgcttacaatttcct 3520
gatgcggtattttctccttacgcatctgtgcggtatttcacaccgcatacgcgga 3575
tctgcgcagcaccatggcctgaaataacctctgaaagaggaacttggttaggtac 3630
cttctgaggcggaaagaaccagctgtggaatgtgtgtcagttagggtgtggaaag 3685
tccccaggctccccagcaggcagaagtatgcaaagcatgcatctcaattagtcag 3740
caaccaggtgtggaaagtccccaggctccccagcaggcagaagtatgcaaagcat 3795
gcatctcaattagtcagcaaccatagtcccgcccctaactccgcccatcccgccc 3850
ctaactccgcccagttccgcccattctccgccccatggctgactaatttttttta 3905
tttatgcagaggccgaggccgcctcggcctctgagctattccagaagtagtgagg 3960
aggcttttttggaggcctaggcttttgcaaaaagcttgattcttctgacacaaca 4015
gtctcgaacttaaggctagagccaccatgattgaacaagatggattgcacgcagg 4070
ttctccggccgcttgggtggagaggctattcggctatgactgggcacaacagaca 4125
atcggctgctctgatgccgccgtgttccggctgtcagcgcaggggcgcccggttc 4180
tttttgtcaagaccgacctgtccggtgccctgaatgaactgcaggacgaggcagc 4235
gcggctatcgtggctggccacgacgggcgttccttgcgcagctgtgctcgacgtt 4290
gtcactgaagcgggaagggactggctgctattgggcgaagtgccggggcaggatc 4345
tcctgtcatctcaccttgctcctgccgagaaagtatccatcatggctgatgcaat 4400
gcggcggctgcatacgcttgatccggctacctgcccattcgaccaccaagcgaaa 4455
catcgcatcgagcgagcacgtactcggatggaagccggtcttgtcgatcaggatg 4510
atctggacgaagagcatcaggggctcgcgccagccgaactgttcgccaggctcaa 4565
ggcgcgcatgcccgacggcgaggatctcgtcgtgacccatggcgatgcctgcttg 4620
ccgaatatcatggtggaaaatggccgcttttctggattcatcgactgtggccggc 4675
tgggtgtggcggaccgctatcaggacatagcgttggctacccgtgatattgctga 4730
agagcttggcggcgaatgggctgaccgcttcctcgtgctttacggtatcgccgct 4785
cccgattcgcagcgcatcgccttctatcgccttcttgacgagttcttctgagcgg 4840
gactctggggttcgaaatgaccgaccaagcgacgcccaacctgccatcacgatgg 4895
ccgcaataaaatatctttattttcattacatctgtgtgttggttttttgtgtgaa 4950
tcgatagcgataaggatccgcgtatggtgcactctcagtacaatctgctctgatg 5005
ccgcatagttaagccagccccgacacccgccaacacccgctgacgcgccctgacg 5060
ggcttgtctgctcccggcatccgcttacagacaagctgtgaccgtctccgggagc 5115
tgcatgtgtcagaggttttcaccgtcatcaccgaaacgcgcgagacgaaagggcc 5170
tcgtgatacgcctatttttataggttaatgtcatgataataatggtttcttagac 5225
gtcaggtggcacttttcggggaaatgtgcgcggaacccctatttgtttatttttc 5280
taaatacattcaaatatgtatccgctcatgagacaataaccctgataaatgcttc 5335
aataatattgaaaaaggaagagtatgagtattcaacatttccgtgtcgcccttat 5390
tcccttttttgcggcattttgccttcctgtttttgctcacccagaaacgctggtg 5445
aaagtaaaagatgctgaagatcagttgggtgcacgagtgggttacatcgaactgg 5500
atctcaacagcggtaagatccttgagagttttcgccccgaagaacgttttccaat 5555
gatgagcacttttaaagttctgctatgtggcgcggtattatcccgtattgacgcc 5610
gggcaagagcaactcggtcgccgcatacactattctcagaatgacttggttgagt 5665
actcaccagtcacagaaaagcatcttacggatggcatgacagtaagagaattatg 5720
cagtgctgccataaccatgagtgataacactgcggccaacttacttctgacaacg 5775
atcggaggaccgaaggagctaaccgcttttttgcacaacatgggggatcatgtaa 5830
ctcgccttgatcgttgggaaccggagctgaatgaagccataccaaacgacgagcg 5885
tgacaccacgatgcctgtagcaatggcaacaacgttgcgcaaactattaactggc 5940
gaactacttactctagcttcccggcaacaattaatagactggatggaggcggata 5995
aagttgcaggaccacttctgcgctcggcccttccggctggctggtttattgctga 6050
taaatctggagccggtgagcgtgggtctcgcggtatcattgcagcactggggcca 6105
gatggtaagccctcccgtatcgtagttatctacacgacggggagtcaggcaacta 6160
tggatgaacgaaatagacagatcgctgagataggtgcctcactgattaagcattg 6215
gtaactgtcagaccaagtttactcatatatactttagattgatttaaaacttcat 6270
tttaatttaaaaggatctaggtgaagatcctttttgataatctcatgaccaaaa 6325
tcccttaacgtgagttttcgttccactgagcgtcagaccccgtagaaaagatcaa 6380
aggatcttcttgagatcctttttttctgcgcgtaatctgctgcttgcaaacaaaa 6435
aaaccaccgctaccagcggtggtttgtttgccggatcaagagctaccaactcttt 6490
ttccgaaggtaactggcttcagcagagcgcagataccaaatactgttcttctagt 6545
gtagccgtagttaggccaccacttcaagaactctgtagcaccgcctacatacctc 6600
```

```
gctctgctaatcctgttaccagtggctgctgccagtggcgataagtcgtgtctta    6655
ccgggttggactcaagacgatagttaccggataaggcgcagcggtcgggctgaac    6710
ggggggttcgtgcacacagcccagcttggagcgaacgacctacaccgaactgaga    6765
tacctacagcgtgagctatgagaaagcgccacgcttcccgaagggagaaaggcgg    6820
acaggtatccggtaagcggcagggtcggaacaggagagcgcacgagggagcttcc    6875
agggggaaacgcctggtatctttatagtcctgtcgggtttcgccacctctgactt    6930
gagcgtcgatttttgtgatgctcgtcaggggggcggagcctatggaaaaacgcca    6985
gcaacgcggcctttttacggttcctggccttttgctggccttttgctcacatggc    7040
tcgacagatct •••   7051
```

DNA Type: Synthetic DNA

Description: Rattus norvegicus gamma-aminobutyric acid type A receptor subunit alpha 1 (Gabra1) with gain-of-function mutation L9'T.

Created: Jul 9, 2024
Last Modified: Nov 12, 2024

Accession Number:
Code Number:

Sequence Author:

Comments: pUNIV vector suitable for mammalian cells and Xenopus laevis oocytes. Stop codon changed to TGA so as not to interfere with nonsense suppression.

References:
1. Venkatachalan SP, Bushman JD, Mercado JL, Sancar F, Christopherson KR, Boileau AJ.
Optimized expression vector for ion channel studies in Xenopus oocytes and mammalian cells using alfalfa mosaic virus.
Pflugers Arch 2007 Apr;454:155-63
PubMed ID: 17146677

Embedded Files:
