# Supplementary material for: A single main-chain hydrogen bond required to keep GABAA receptors closed
Source: Nat Commun. 2025 Jul 3;16:6107. doi: 10.1038/s41467-025-61447-0 (PMC12222489; doi:10.1038/s41467-025-61447-0)
Supplement: Supplementary file 5 — Supplementary Data 3 [file 41467_2025_61447_MOESM5_ESM.pdf]

Sequence: pUNIV-rat Gabra1(L263T-V279tag).dna (Circular / 7051 bp)
Enzymes: Unique 6+ Cutters (42 of 666 total)
Features: 16 total

Unique Cutters **Bold**

Rattus norvegicus gamma-aminobutyric acid type A receptor subunit alpha 1 (Gabra1) with gain-of-function mutation L9'T with stop codon Val279tag for nonsense suppression substitution of unnatural amino acids.

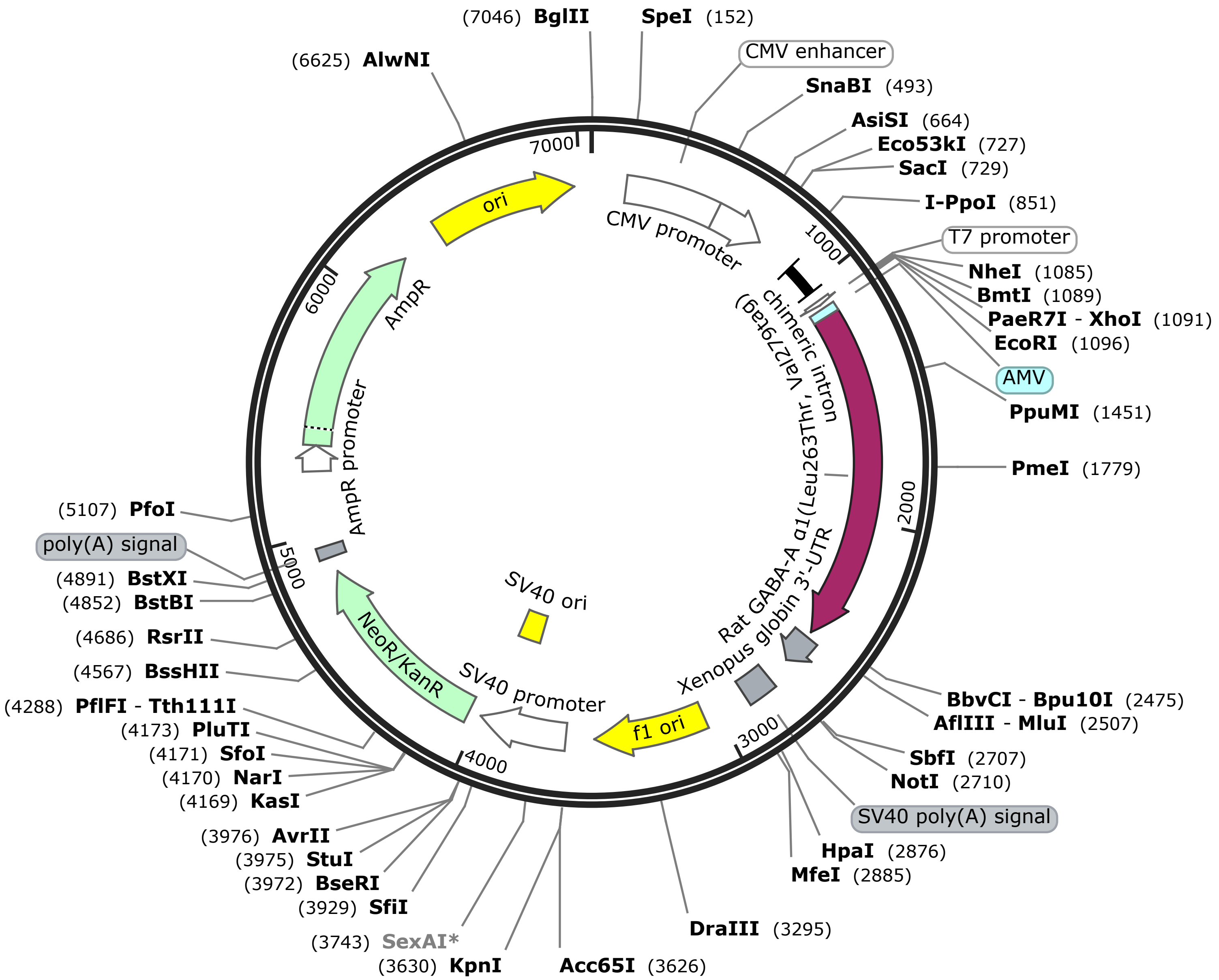

pUNIV-rat Gabra1(L263T-V279tag)
7051 bp

```
••• tcaatattggccattagccatattattcattggttatatagcataaatcaatatt   55
    ggctattggccattgcatacgttgtatctatatcataatatgtacatttatattg   110
    gctcatgtccaatatgaccgccatgttggcattgattattgactagttattaata   165
    gtaatcaattacggggtcattagttcatagcccatatatggagttccgcgttaca   220
    taacttacggtaaatggcccgcctggctgaccgcccaacgacccccgcccattga   275
    cgtcaataatgacgtatgttcccatagtaacgccaatagggactttccattgacg   330
    tcaatgggtggagtatttacggtaaactgcccacttggcagtacatcaagtgtat   385
    catatgccaagtccgccccctattgacgtcaatgacggtaaatggcccgcctggc   440
    attatgcccagtacatgaccttacgggactttcctacttggcagtacatctacgt   495
    attagtcatcgctattaccatggtgatgcggttttggcagtacaccaatgggcgt   550
    ggatagcggtttgactcacggggatttccaagtctccaccccattgacgtcaatg   605
    ggagtttgttttggcaccaaaatcaacgggactttccaaaatgtcgtaacaactg   660
    cgatcgcccgccccgttgacgcaaatgggcggtaggcgtgtacggtgggaggtct   715
    atataagcagagctcgtttagtgaaccgtcagatcactagaagctttattgcggt   770
    agtttatcacagttaaattgctaacgcagtcagtgcttctgacacaacagtctcg   825
    aacttaagctgcagtgactctcttaaggtagccttgcagaagttggtcgtgaggc   880
    actgggcaggtaagtatcaaggttacaagacaggtttaaggagaccaatagaaac   935
    tgggcttgtcgagacagagaagactcttgcgtttctgataggcacctattggtct   990
    tactgacatccactttgcctttctctccacaggtgtccactcccagttcaattac   1045
    agctcttaaggctagagtacttaatacgactcactataggctagcctcgagaatt   1100
    ccgttttatttttaatttttctttcaaatacttccaccatgaagaaaagtcgggg   1155
    tctctctgactatctttgggcctggaccctcattctgagcactctctcgggaaga   1210
    agctatggacagccctcccaagatgaacttaaggacaacaccactgtcttcacga   1265
    ggattttggaccgactgctggatggttatgacaatcgtctgagaccaggcttggg   1320
    agagcgtgtaactgaagtgaagacggacatctttgtcaccagtttcggacccgtg   1375
    tcagaccacgatatggaatatacaatagatgtgtttttccgccaaagctggaagg   1430
    atgaaagattaaaattcaaaggacccatgacagtgctccggctgaacaacctgat   1485
    ggccagtaaaatctggactccagatacatttttccacaatggaaaaaagtctgtg   1540
    gcccacaacatgaccatgcccaataaactcctgcgtatcacagaggatggcacac   1595
    tgctgtacaccatgaggttgactgtgagagccgaatgccccatgcacttagaaga   1650
    ctttcccatggatgcccatgcctgcccactaaaatttgggagctatgcttataca   1705
    agagcagaagttgtctatgagtggacaagggagccagcccgctcagtggttgtgg   1760
    cagaagatgggtcacgtttaaaccagtatgaccttcttgggcaaacagttgactc   1815
    tggaattgttcagtccagtactggagaatatgtggttatgacgactcactttcac   1870
    ttgaagagaaaaatcggctactttgttattcaaacatatctgccatgcataatga   1925
    cagtcattctctcccaagtctccttctggcttaacagagagtcagtaccagcaag   1980
    aactgtctttggagtgacgaccgttaccaccatgacaaccttgagtatcagtgcc   2035
    agaaattccctcccaaagtaggcttatgcaacggccatggactggtttattgcag   2090
    tgtgctatgccttcgtgttctcggctctgattgagtttgccacagtaaactattt   2145
    caccaagagagggtatgcgtgggatggcaaaagcgtggttccagaaaagccaaag   2200
    aaagtgaaggatcctctcattaagaaaaacaacacatatgctcctacagcaacca   2255
    gctataccccctaacttagccaggggtgaccccggcttggcaactattgctaaaag   2310
    tgcgaccatagaaccgaaagaagtcaagcctgagacaaaaccgccagaacccaag   2365
    aaaacctttaacagcgtcagcaaaatcgaccgactgtcaagaatagcctttccgc   2420
    tgctatttggaatctttaacttagtctattgggccacgtatttaaacagagagcc   2475
    tcagctaaaagcccccacaccccatcaatgaacgcgtgatctggttaccactaaa   2530
    ccagcctcaagaacacccgaatggagtctctaagctacataataccaacttacac   2585
    tttacaaaatgttgtcccccaaaatgtagccattcgtatctgctcctaataaaaa   2640
    gaaagtttcttcacattctaaaaaaaaaaaaaaaaaaaaaaaaaaaaaaaaaccc   2695
    cccccccctgcaggcggccgcttccctttagtgagggttaatgcttcgagcagac   2750
    atgataagatacattgatgagtttggacaaaccacaactagaatgcagtgaaaaa   2805
    aatgctttatttgtgaaatttgtgatgctattgctttatttgtaaccattataag   2860
    ctgcaataaacaagttaacaacaacaattgcattcattttatgtttcaggttcag   2915
    ggggagatgtgggaggttttttaaagcaagtaaaacctctacaaatgtggtaaaa   2970
    tccgataaggatcgatccgggctggcgtaatagcgaagaggcccgcaccgatcgc   3025
    ccttcccaacagttgcgcagcctgaatggcgaatggacgcgccctgtagcggcgc   3080
    attaagcgcggcgggtgtggtggttacgcgcagcgtgaccgctacacttgccagc   3135
    gccctagcgcccgctcctttcgctttcttcccttcctttctcgccacgttcgccg   3190
    gctttccccgtcaagctctaaatcgggggctccctttagggttccgatttagtgc   3245
    tttacggcacctcgaccccaaaaaacttgattagggtgatggttcacgtagtggg   3300
```

```
ccatcgccctgatagacggtttttcgccctttgacgttggagtccacgttcttta 3355
atagtggactcttgttccaaactggaacaacactcaaccctatctcggtctattc 3410
ttttgatttataagggattttgccgatttcggcctattggttaaaaaatgagctg 3465
atttaacaaaaatttaacgcgaattttaacaaaatattaacgcttacaatttcct 3520
gatgcggtattttctccttacgcatctgtgcggtatttcacaccgcatacgcgga 3575
tctgcgcagcaccatggcctgaaataacctctgaaagaggaacttggttaggtac 3630
cttctgaggcggaaagaaccagctgtggaatgtgtgtcagttagggtgtggaaag 3685
tccccaggctccccagcaggcagaagtatgcaaagcatgcatctcaattagtcag 3740
caaccaggtgtggaaagtccccaggctccccagcaggcagaagtatgcaaagcat 3795
gcatctcaattagtcagcaaccatagtcccgcccctaactccgcccatcccgccc 3850
ctaactccgcccagttccgcccattctccgccccatggctgactaatttttttta 3905
tttatgcagaggccgaggccgcctcggcctctgagctattccagaagtagtgagg 3960
aggcttttttggaggcctaggcttttgcaaaaagcttgattcttctgacacaaca 4015
gtctcgaacttaaggctagagccaccatgattgaacaagatggattgcacgcagg 4070
ttctccggccgcttgggtggagaggctattcggctatgactgggcacaacagaca 4125
atcggctgctctgatgccgccgtgttccggctgtcagcgcaggggcgcccggttc 4180
tttttgtcaagaccgacctgtccggtgccctgaatgaactgcaggacgaggcagc 4235
gcggctatcgtggctggccacgacgggcgttccttgcgcagctgtgctcgacgtt 4290
gtcactgaagcgggaagggactggctgctattgggcgaagtgccggggcaggatc 4345
tcctgtcatctcaccttgctcctgccgagaaagtatccatcatggctgatgcaat 4400
gcggcggctgcatacgcttgatccggctacctgcccattcgaccaccaagcgaaa 4455
catcgcatcgagcgagcacgtactcggatggaagccggtcttgtcgatcaggatg 4510
atctggacgaagagcatcaggggctcgcgccagccgaactgttcgccaggctcaa 4565
ggcgcgcatgcccgacggcgaggatctcgtcgtgacccatggcgatgcctgcttg 4620
ccgaatatcatggtggaaaatggccgcttttctggattcatcgactgtggccggc 4675
tgggtgtggcggaccgctatcaggacatagcgttggctacccgtgatattgctga 4730
agagcttggcggcgaatgggctgaccgcttcctcgtgctttacggtatcgccgct 4785
cccgattcgcagcgcatcgccttctatcgccttcttgacgagttcttctgagcgg 4840
gactctggggttcgaaatgaccgaccaagcgacgcccaacctgccatcacgatgg 4895
ccgcaataaaatatctttattttcattacatctgtgtgttggtttttgtgtgaa 4950
tcgatagcgataaggatccgcgtatggtgcactctcagtacaatctgctctgatg 5005
ccgcatagttaagccagccccgacacccgccaacacccgctgacgcgccctgacg 5060
ggcttgtctgctcccggcatccgcttacagacaagctgtgaccgtctccgggagc 5115
tgcatgtgtcagaggttttcaccgtcatcaccgaaacgcgcgagacgaaagggcc 5170
tcgtgatacgcctatttttataggttaatgtcatgataataatggtttcttagac 5225
gtcaggtggcacttttcggggaaatgtgcgcggaacccctatttgtttatttttc 5280
taaatacattcaaatatgtatccgctcatgagacaataaccctgataaatgcttc 5335
aataatattgaaaaaggaagagtatgagtattcaacatttccgtgtcgcccttat 5390
tcccttttttgcggcattttgccttcctgtttttgctcacccagaaacgctggtg 5445
aaagtaaaagatgctgaagatcagttgggtgcacgagtgggttacatcgaactgg 5500
atctcaacagcggtaagatccttgagagttttcgccccgaagaacgttttccaat 5555
gatgagcacttttaaagttctgctatgtggcgcggtattatcccgtattgacgcc 5610
gggcaagagcaactcggtcgccgcatacactattctcagaatgacttggttgagt 5665
actcaccagtcacagaaaagcatcttacggatggcatgacagtaagagaattatg 5720
cagtgctgccataaccatgagtgataacactgcggccaacttacttctgacaacg 5775
atcggaggaccgaaggagctaaccgcttttttgcacaacatgggggatcatgtaa 5830
ctcgccttgatcgttgggaaccggagctgaatgaagccataccaaacgacgagcg 5885
tgacaccacgatgcctgtagcaatggcaacaacgttgcgcaaactattaactggc 5940
gaactacttactctagcttcccggcaacaattaatagactggatggaggcggata 5995
aagttgcaggaccacttctgcgctcggcccttccggctggctggtttattgctga 6050
taaatctggagccggtgagcgtgggtctcgcggtatcattgcagcactggggcca 6105
gatggtaagccctcccgtatcgtagttatctacacgacggggagtcaggcaacta 6160
tggatgaacgaaatagacagatcgctgagataggtgcctcactgattaagcattg 6215
gtaactgtcagaccaagtttactcatatatactttagattgatttaaaacttcat 6270
tttaatttaaaaggatctaggtgaagatcctttttgataatctcatgaccaaaa 6325
tcccttaacgtgagttttcgttccactgagcgtcagaccccgtagaaaagatcaa 6380
aggatcttcttgagatcctttttttctgcgcgtaatctgctgcttgcaaacaaaa 6435
aaaccaccgctaccagcggtggtttgtttgccggatcaagagctaccaactcttt 6490
ttccgaaggtaactggcttcagcagagcgcagataccaaatactgttcttctagt 6545
gtagccgtagttaggccaccacttcaagaactctgtagcaccgcctacatacctc 6600
```

gctctgctaatcctgttaccagtggctgctgccagtggcgataagtcgtgtctta 6655
ccgggttggactcaagacgatagttaccggataaggcgcagcggtcgggctgaac 6710
ggggggttcgtgcacacagcccagcttggagcgaacgacctacaccgaactgaga 6765
tacctacagcgtgagctatgagaaagcgccacgcttcccgaagggagaaaggcgg 6820
acaggtatccggtaagcggcagggtcggaacaggagagcgcacgagggagcttcc 6875
agggggaaacgcctggtatctttatagtcctgtcgggtttcgccacctctgactt 6930
gagcgtcgatttttgtgatgctcgtcaggggggcggagcctatggaaaaacgcca 6985
gcaacgcggcctttttacggttcctggccttttgctggccttttgctcacatggc 7040
tcgacagatct ••• 7051

DNA Type: Synthetic DNA

Description: Rattus norvegicus gamma-aminobutyric acid type A receptor subunit alpha 1 (Gabra1) with gain-of-function mutation L9'T with stop codon Val279tag for nonsense suppression substitution of unnatural amino acids.

Created: Jul 9, 2024
Last Modified: Nov 12, 2024

Accession Number:
Code Number:

Sequence Author:

Comments: pUNIV vector suitable for mammalian cells and Xenopus laevis oocytes. Stop codon changed to TGA so as not to interfere with nonsense suppression.

References:
1. Venkatachalan SP, Bushman JD, Mercado JL, Sancar F, Christopherson KR, Boileau AJ.
Optimized expression vector for ion channel studies in Xenopus oocytes and mammalian cells using alfalfa mosaic virus.
Pflugers Arch 2007 Apr;454:155-63
PubMed ID: 17146677

Embedded Files:
