# Supplementary material for: A single main-chain hydrogen bond required to keep GABAA receptors closed
Source: Nat Commun. 2025 Jul 3;16:6107. doi: 10.1038/s41467-025-61447-0 (PMC12222489; doi:10.1038/s41467-025-61447-0)
Supplement: Supplementary file 6 — Supplementary Data 4 [file 41467_2025_61447_MOESM6_ESM.pdf]

Sequence: pUNIV-rat Gabrb2.dna (Circular / 7108 bp)
Enzymes: Unique 6+ Cutters (50 of 666 total)
Features: 18 total

Unique Cutters **Bold**

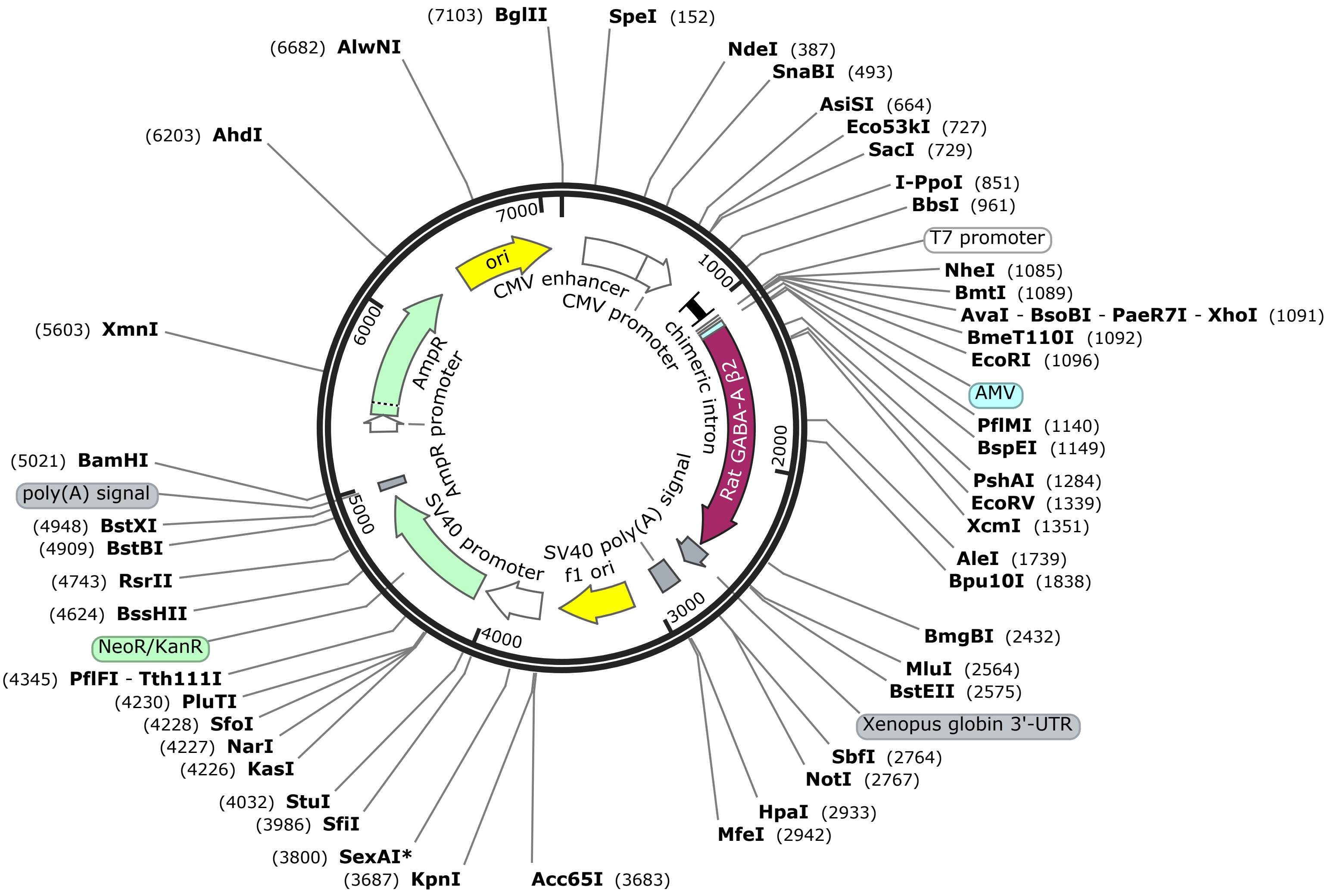

pUNIV-rat Gabrb2
7108 bp

```
••• tcaatattggccattagccatattattcattggttatatagcataaatcaatatt 55
ggctattggccattgcatacgttgtatctatatcataatatgtacatttatattg 110
gctcatgtccaatatgaccgccatgttggcattgattattgactagttattaata 165
gtaatcaattacggggtcattagttcatagcccatatatggagttccgcgttaca 220
taacttacggtaaatggcccgcctggctgaccgcccaacgacccccgcccattga 275
cgtcaataatgacgtatgttcccatagtaacgccaatagggactttccattgacg 330
tcaatgggtggagtatttacggtaaactgcccacttggcagtacatcaagtgtat 385
catatgccaagtccgcccctattgacgtcaatgacggtaaatggcccgcctggc 440
attatgcccagtacatgaccttacgggactttcctacttggcagtacatctacgt 495
attagtcatcgctattaccatggtgatgcggttttggcagtacaccaatgggcgt 550
ggatagcggtttgactcacggggatttccaagtctccaccccattgacgtcaatg 605
ggagtttgttttggcaccaaaatcaacgggactttccaaaatgtcgtaacaactg 660
cgatcgcccgccccgttgacgcaaatgggcggtaggcgtgtacggtgggaggtct 715
atataagcagagctcgtttagtgaaccgtcagatcactagaagctttattgcggt 770
agtttatcacagttaaattgctaacgcagtcagtgcttctgacacaacagtctcg 825
aacttaagctgcagtgactctcttaaggtagccttgcagaagttggtcgtgaggc 880
actgggcaggtaagtatcaaggttacaagacaggtttaaggagaccaatagaaac 935
tgggcttgtcgagacagagaagactcttgcgtttctgataggcacctattggtct 990
tactgacatccactttgcctttctctccacaggtgtccactcccagttcaattac 1045
agctcttaaggctagagtacttaatacgactcactataggctagcctcgagaatt 1100
ccgttttatttttaatttctttcaaatacttccaccatgtggagagtccggaa 1155
aaggggctactttgggatttggtcatttcccttaataatcgccgctgtctgtgct 1210
cagagtgtcaatgaccctagtaatatgtcgctggttaaagagacggtggacagac 1265
tgttgaaaggctatgacattcgtctgagaccagatttcggaggtccccctgtggc 1320
agtaggaatgaacattgatatcgccagcatcgatatggtttctgaagtcaatatg 1375
gactacaccttgaccatgtatttccagcaagcctggagagataagagactgtcct 1430
acaatgtaatccctttaaacttgactttggacaatcgagtggcagaccagctctg 1485
ggtgcctgacacctacttcctgaatgataagaagtcatttgtacatggagtgact 1540
gtcaaaaaccgtatgattcgactgcatccagatggtactgtcctgtatggcctca 1595
gaatcacaactacagctgcctgcatgatggacctaaggcggtatccactggatga 1650
acaaaactgcacgttggagatcgaaagctatggctatacaactgatgacattgag 1705
ttttactggcgtggcgatgacaatgcagtcacgggagtgacaaagattgagcttc 1760
ctcagttctccattgtagattataaactcatcaccaagaaagttgttttctccac 1815
aggttcttatcccagattgtccctaagctttaagctgaaaagaaacattggctac 1870
ttcatcctgcagacatacatgccatccattctgattaccatcctctcctgggtct 1925
cctttttggatcaactatgatgcttctgctgcacgggttgcattaggaattacaac 1980
tgtcctgacgatgaccacaatcaatacccatctccgggagactctccctaaaatt 2035
ccctatgtaaaagccattgacatgtacctaatggggtgctttgtctttgtcttta 2090
tggcccttctggaatatgctttggtcaactacatcttctttgggagaggacccca 2145
gcgccaaaagaaagcagctgagaaagctgctaatgccaacaacgagaagatgcgc 2200
ctggatgtcaacaagatggacccacatgagaacatcttactcagcactcttgaga 2255
taaaaaatgagatggccacatcagaagcagtaatgggacttggagaccccaggag 2310
cacaatgcttgcctatgatgcctccagcatccagtatcggaaagctgggttgcct 2365
aggcatagttttggccgcaacgccctggaacgacatgtggcacaaaagaaaagtc 2420
gcctgaggagacgtgcctcccaactgaaaatcaccatccccgacttgactgatgt 2475
gaacgccattgatcggtggtcccgcattttcttccctgtggtgttttccttcttc 2530
aacatcgtctattggctttactatgtgaactaaacgcgtgatctggttaccacta 2585
aaccagcctcaagaacacccgaatggagtctctaagctacataataccaacttac 2640
actttacaaaatgttgtcccccaaaatgtagccattcgtatctgctcctaataaa 2695
aagaaagtttcttcacattctaaaaaaaaaaaaaaaaaaaaaaaaaaaaaaaaac 2750
ccccccccctgcaggcggccgcttccctttagtgagggttaatgcttcgagcag 2805
acatgataagatacattgatgagtttggacaaaccacaactagaatgcagtgaaa 2860
aaaatgctttatttgtgaaatttgtgatgctattgctttatttgtaaccattata 2915
agctgcaataaacaagttaacaacaacaattgcattcattttatgtttcaggttc 2970
agggggagatgtgggaggttttttaaagcaagtaaaacctctacaaatgtggtaa 3025
aatccgataaggatcgatccgggctggcgtaatagcgaagaggcccgcaccgatc 3080
gcccttcccaacagttgcgcagcctgaatggcgaatggacgcgccctgtagcggc 3135
gcattaagcgcggcgggtgtggtggttacgcgcagcgtgaccgctacacttgcca 3190
gcgccctagcgcccgctcctttcgctttcttcccttcctttctcgccacgttcgc 3245
cggctttccccgtcaagctctaaatcgggggctccctttagggttccgatttagt 3300
```

gctttacggcacctcgaccccaaaaaacttgattagggtgatggttcacgtagtg 3355
ggccatcgccctgatagacggtttttcgccctttgacgttggagtccacgttctt 3410
taatagtggactcttgttccaaactggaacaacactcaaccctatctcggtctat 3465
tcttttgatttataagggattttgccgatttcggcctattggttaaaaaatgagc 3520
tgatttaacaaaaatttaacgcgaattttaacaaaatattaacgcttacaatttc 3575
ctgatgcggtattttctccttacgcatctgtgcggtatttcacaccgcatacgcg 3630
gatctgcgcagcaccatggcctgaaataacctctgaaagaggaacttggttaggt 3685
accttctgaggcggaaagaaccagctgtggaatgtgtgtcagttagggtgtggaa 3740
agtccccaggctccccagcaggcagaagtatgcaaagcatgcatctcaattagtc 3795
agcaaccaggtgtggaaagtccccaggctccccagcaggcagaagtatgcaaagc 3850
atgcatctcaattagtcagcaaccatagtcccgcccctaactccgcccatcccgc 3905
ccctaactccgcccagttccgcccattctccgccccatggctgactaattttttt 3960
tatttatgcagaggccgaggccgcctcggcctctgagctattccagaagtagtga 4015
ggaggcttttttggaggcctaggcttttgcaaaaagcttgattcttctgacacaa 4070
cagtctcgaacttaaggctagagccaccatgattgaacaagatggattgcacgca 4125
ggttctccggccgcttgggtggagaggctattcggctatgactgggcacaacaga 4180
caatcggctgctctgatgccgccgtgttccggctgtcagcgcaggggcgcccggt 4235
tctttttgtcaagaccgacctgtccggtgccctgaatgaactgcaggacgaggca 4290
gcgcggctatcgtggctggccacgacgggcgttccttgcgcagctgtgctcgacg 4345
ttgtcactgaagcgggaagggactggctgctattgggcgaagtgccggggcagga 4400
tctcctgtcatctcaccttgctcctgccgagaaagtatccatcatggctgatgca 4455
atgcggcggctgcatacgcttgatccggctacctgcccattcgaccaccaagcga 4510
aacatcgcatcgagcgagcacgtactcggatggaagccggtcttgtcgatcagga 4565
tgatctggacgaagagcatcaggggctcgcgccagccgaactgttcgccaggctc 4620
aaggcgcgcatgcccgacggcgaggatctcgtcgtgacccatggcgatgcctgct 4675
tgccgaatatcatggtggaaaatggccgcttttctggattcatcgactgtggccg 4730
gctgggtgtggcggaccgctatcaggacatagcgttggctacccgtgatattgct 4785
gaagagcttggcggcgaatgggctgaccgcttcctcgtgctttacggtatcgccg 4840
ctcccgattcgcagcgcatcgccttctatcgccttcttgacgagttcttctgagc 4895
gggactctggggttcgaaatgaccgaccaagcgacgcccaacctgccatcacgat 4950
ggccgcaataaaatatctttattttcattacatctgtgtgttggttttttgtgtg 5005
aatcgatagcgataaggatccgcgtatggtgcactctcagtacaatctgctctga 5060
tgccgcatagttaagccagccccgacacccgccaacacccgctgacgcgccctga 5115
cgggcttgtctgctcccggcatccgcttacagacaagctgtgaccgtctccggga 5170
gctgcatgtgtcagaggttttcaccgtcatcaccgaaacgcgcgagacgaaaggg 5225
cctcgtgatacgcctatttttataggttaatgtcatgataataatggtttcttag 5280
acgtcaggtggcacttttcggggaaatgtgcgcggaacccctatttgtttatttt 5335
tctaaatacattcaaatatgtatccgctcatgagacaataaccctgataaatgct 5390
tcaataatattgaaaaaggaagagtatgagtattcaacatttccgtgtcgccctt 5445
attcccttttttgcggcattttgccttcctgtttttgctcacccagaaacgctgg 5500
tgaaagtaaaagatgctgaagatcagttgggtgcacgagtgggttacatcgaact 5555
ggatctcaacagcggtaagatccttgagagttttcgccccgaagaacgttttcca 5610
atgatgagcacttttaaagttctgctatgtggcgcggtattatcccgtattgacg 5665
ccgggcaagagcaactcggtcgccgcatacactattctcagaatgacttggttga 5720
gtactcaccagtcacagaaaagcatcttacggatggcatgacagtaagagaatta 5775
tgcagtgctgccataaccatgagtgataacactgcggccaacttacttctgacaa 5830
cgatcggaggaccgaaggagctaaccgcttttttgcacaacatgggggatcatgt 5885
aactcgccttgatcgttgggaaccggagctgaatgaagccataccaaacgacgag 5940
cgtgacaccacgatgcctgtagcaatggcaacaacgttgcgcaaactattaactg 5995
gcgaactacttactctagcttcccggcaacaattaatagactggatggaggcgga 6050
taaagttgcaggaccacttctgcgctcggcccttccggctggctggtttattgct 6105
gataaatctggagccggtgagcgtgggtctcgcggtatcattgcagcactggggc 6160
cagatggtaagccctcccgtatcgtagttatctacacgacggggagtcaggcaac 6215
tatggatgaacgaaatagacagatcgctgagataggtgcctcactgattaagcat 6270
tggtaactgtcagaccaagtttactcatatatactttagattgatttaaaacttc 6325
atttttaatttaaaaggatctaggtgaagatcctttttgataatctcatgaccaa 6380
aatcccttaacgtgagttttcgttccactgagcgtcagaccccgtagaaaagatc 6435
aaaggatcttcttgagatcctttttttctgcgcgtaatctgctgcttgcaaacaa 6490
aaaaaccaccgctaccagcggtggtttgtttgccggatcaagagctaccaactct 6545
tttccgaaggtaactggcttcagcagagcgcagataccaaatactgttcttcta 6600

```
gtgtagccgtagttaggccaccacttcaagaactctgtagcaccgcctacatacc     6655
tcgctctgctaatcctgttaccagtggctgctgccagtggcgataagtcgtgtct     6710
taccgggttggactcaagacgatagttaccggataaggcgcagcggtcgggctga     6765
acggggggttcgtgcacacagcccagcttggagcgaacgacctacaccgaactga     6820
gatacctacagcgtgagctatgagaaagcgccacgcttcccgaagggagaaaggc     6875
ggacaggtatccggtaagcggcagggtcggaacaggagagcgcacgagggagctt     6930
ccagggggaaacgcctggtatctttatagtcctgtcgggtttcgccacctctgac     6985
ttgagcgtcgatttttgtgatgctcgtcaggggggcggagcctatggaaaaacgc     7040
cagcaacgcggcctttttacggttcctggccttttgctggccttttgctcacatg     7095
gctcgacagatct ••• 7108
```

DNA Type: Synthetic DNA

Description: Rattus norvegicus gamma-aminobutyric acid type A receptor subunit beta 2 (Gabrb2)

Created: Apr 10, 2024
Last Modified: Nov 15, 2024

Accession Number: NM_012957.3
Code Number:

Sequence Author: Goldschen Lab

Comments: pUNIV vector suitable for mammalian cells and Xenopus laevis oocytes.

References:
1. Venkatachalan SP, Bushman JD, Mercado JL, Sancar F, Christopherson KR, Boileau AJ.
Optimized expression vector for ion channel studies in Xenopus oocytes and mammalian cells using alfalfa mosaic virus.
Pflugers Arch 2007 Apr;454:155-63
PubMed ID: 17146677

Embedded Files:
