# Supplementary material for: A single main-chain hydrogen bond required to keep GABAA receptors closed
Source: Nat Commun. 2025 Jul 3;16:6107. doi: 10.1038/s41467-025-61447-0 (PMC12222489; doi:10.1038/s41467-025-61447-0)
Supplement: Supplementary file 8 — Supplementary Data 6 [file 41467_2025_61447_MOESM8_ESM.pdf]

Sequence: pUNIV-rat Gabrg2.dna (Circular / 7115 bp)
Enzymes: Unique 6+ Cutters (39 of 666 total)
Features: 18 total

Unique Cutters **Bold**

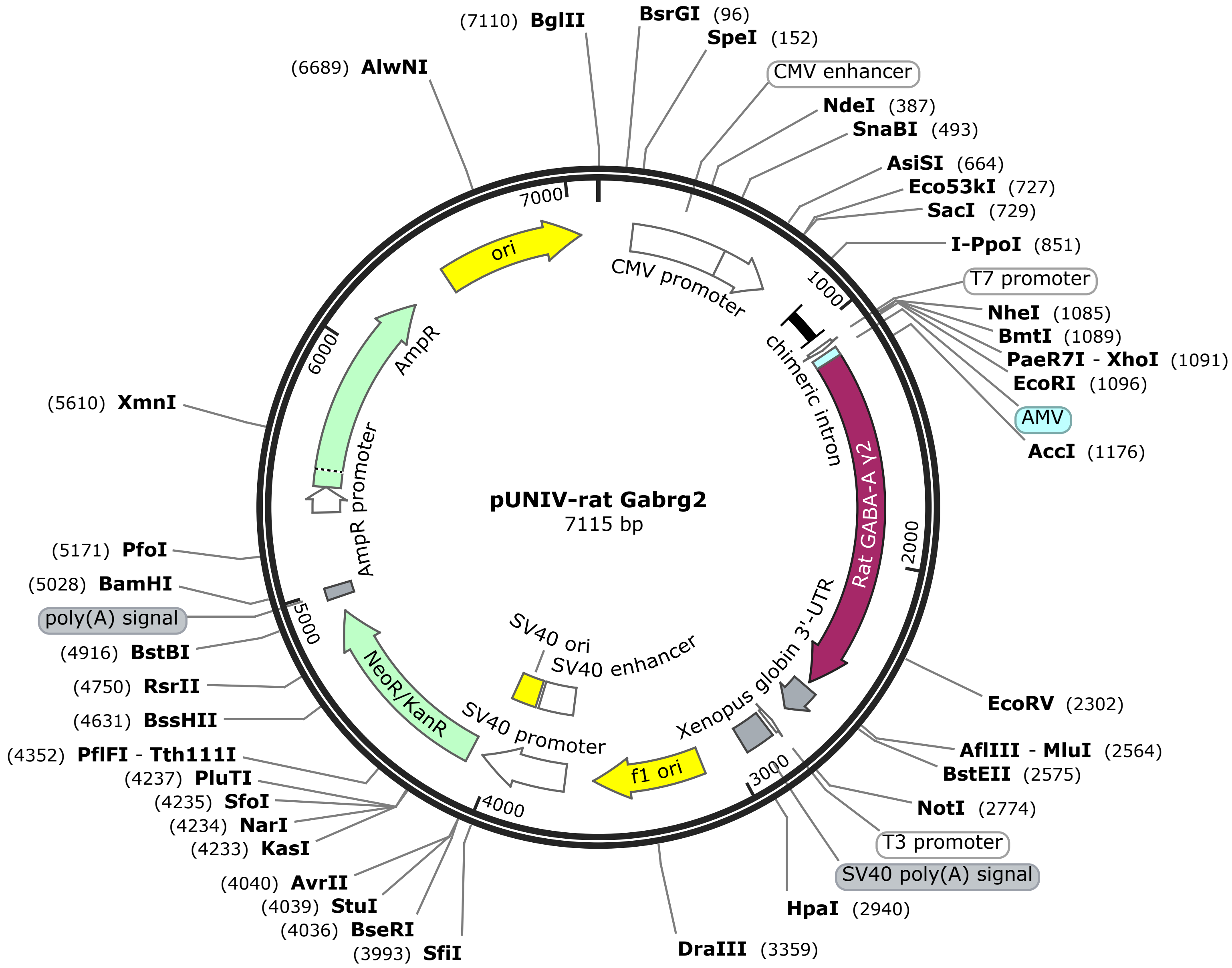

```
••• tcaatattggccattagccatattattcattggttatatagcataaatcaatatt   55
ggctattggccattgcatacgttgtatctatatcataatatgtacatttatattg   110
gctcatgtccaatatgaccgccatgttggcattgattattgactagttattaata   165
gtaatcaattacggggtcattagttcatagcccatatatggagttccgcgttaca   220
taacttacggtaaatggcccgcctggctgaccgcccaacgacccccgcccattga   275
cgtcaataatgacgtatgttcccatagtaacgccaatagggactttccattgacg   330
tcaatgggtggagtatttacggtaaactgcccacttggcagtacatcaagtgtat   385
catatgccaagtccgccccctattgacgtcaatgacggtaaatggcccgcctggc   440
attatgcccagtacatgaccttacgggactttcctacttggcagtacatctacgt   495
attagtcatcgctattaccatggtgatgcggttttggcagtacaccaatgggcgt   550
ggatagcggtttgactcacggggatttccaagtctccaccccattgacgtcaatg   605
ggagtttgttttggcaccaaaatcaacgggactttccaaaatgtcgtaacaactg   660
cgatcgcccgccccgttgacgcaaatgggcggtaggcgtgtacggtgggaggtct   715
atataagcagagctcgtttagtgaaccgtcagatcactagaagctttattgcggt   770
agtttatcacagttaaattgctaacgcagtcagtgcttctgacacaacagtctcg   825
aacttaagctgcagtgactctcttaaggtagccttgcagaagttggtcgtgaggc   880
actgggcaggtaagtatcaaggttacaagacaggtttaaggagaccaatagaaac   935
tgggcttgtcgagacagagaagactcttgcgtttctgataggcacctattggtct   990
tactgacatccactttgcctttctctccacaggtgtccactcccagttcaattac   1045
agctcttaaggctagagtacttaatacgactcactataggctagcctcgagaatt   1100
ccgttttattttaatttctttcaaatacttccaccatgagttcgccaaatac   1155
atggagcactggaagcacagtctactctcctgtattttcacagaaaatgacgctg   1210
tggattctgctcctgctatcgctctacccaggcttcactagccaaaagtcagatg   1265
atgactatgaagattatgcttctaataaaacatgggtgttgactccaaaagttcc   1320
cgagggtgatgtcactgtcatcttaaacaaccttctggaagggtacgacaacaaa   1375
cttcggcccgacataggagtgaaaccaacattaattcatacagatatgtacgtga   1430
acagcattggtccagtgaatgctatcaatatggaatacacaattgatattttttt   1485
tgcccaaacctggtatgacagacgtttgaaatttaacagtaccattaaagttctc   1540
cgattgaatagcaatatggtggggaaaatctggattccagacactttcttcagga   1595
actccaaaaaagcggatgctcactggatcacgactcccaacaggatgctgagaat   1650
ttggaatgacggtcgagttctctacaccttaaggctaacaattgatgctgagtgc   1705
cagttgcaattacacaacttcccaatggatgaacactcctgccccctggagttct   1760
ccagttatggttatcctcgtgaagaaattgtttatcaatggaagcgcagttctgt   1815
tgaagtgggagacacaaggtcatggaggctgtatcagttttcctttgttggattg   1870
aggaataccactgaagtagtgaagacaacttctggtgactatgtggttatgtccg   1925
tgtactttgatctgagcagaagaatggggtactttaccatccagacctacattcc   1980
ctgcacactcattgtggttctgtcctgggtatccttctggatcaataaggatgct   2035
gtccctgcaagaacatctttaggaatcacgactgtcctgacgatgaccactctca   2090
gcaccatagcccggaagtctctgcccaaggtctcctatgtcacagcaatggatct   2145
cttcgtctctgtttgcttcatctttgtgttttcagctttggtggagtatggtacc   2200
ctgcactattttgtgagcaaccggaaaccaagcaaggataaagacaaaaagaaga   2255
aaaaccctcttcttcggatgttttccttcaaggcccctaccattgatatccgtcc   2310
cagatcagcaacgatccaaatgaacaatgccacccaccttcaagagagggatgaa   2365
gaatatggctatgagtgtttggatggcaaggactgtgccagtttcttttgctgtt   2420
ttgaagactgccgaacaggagcctggagacacgggaggatacacattcgcattgc   2475
caaaatggactcctatgctcggatcttcttccctaccgccttctgcttgttcaat   2530
cttgtttactgggtctcctatctttatctgtgaacgcgtgatctggttaccacta   2585
aaccagcctcaagaacacccgaatggagtctctaagctacataataccaacttac   2640
actttacaaaatgttgtccccaaaatgtagccattcgtatctgctcctaataaa   2695
aagaaagtttcttcacattctaaaaaaaaaaaaaaaaaaaaaaaaaaaaaaaaac   2750
ccccccccccccccccctgcagcggccgcttccctttagtgagggttaatgctt   2805
cgagcagacatgataagatacattgatgagtttggacaaaccacaactagaatgc   2860
agtgaaaaaaatgctttatttgtgaaatttgtgatgctattgctttatttgtaac   2915
cattataagctgcaataaacaagttaacaacaacaattgcattcattttatgttt   2970
caggttcagggggagatgtgggaggttttttaaagcaagtaaaacctctacaaat   3025
gtggtaaaatccgataaggatcgatccgggctggcgtaatagcgaagaggcccgc   3080
accgatcgcccttcccaacagttgcgcagcctgaatggcgaatggacgcgccctg   3135
tagcggcgcattaagcgcgggcgggtgtggtggttacgcgcagcgtgaccgctaca   3190
cttgccagcgccctagcgcccgctcctttcgctttcttcccttcctttctcgcca   3245
cgttcgccggctttccccgtcaagctctaaatcgggggctccctttagggttccg   3300
```

atttagtgctttacggcacctcgaccccaaaaaacttgattagggtgatggttca 3355
cgtagtgggccatcgccctgatagacggtttttcgccctttgacgttggagtcca 3410
cgttctttaatagtggactcttgttccaaactggaacaacactcaaccctatctc 3465
ggtctattcttttgatttataagggattttgccgatttcggcctattggttaaaa 3520
aatgagctgatttaacaaaaatttaacgcgaattttaacaaaatattaacgctta 3575
caatttcctgatgcggtattttctccttacgcatctgtgcggtatttcacaccgc 3630
atacgcggatctgcgcagcaccatggcctgaaataacctctgaaagaggaacttg 3685
gttaggtaccttctgaggcggaaagaaccagctgtggaatgtgtgtcagttaggg 3740
tgtggaaagtccccaggctccccagcaggcagaagtatgcaaagcatgcatctca 3795
attagtcagcaaccaggtgtggaaagtccccaggctccccagcaggcagaagtat 3850
gcaaagcatgcatctcaattagtcagcaaccatagtcccgcccctaactccgccc 3905
atcccgcccctaactccgcccagttccgcccattctccgccccatggctgactaa 3960
ttttttttatttatgcagaggccgaggccgcctcggcctctgagctattccagaa 4015
gtagtgaggaggcttttttggaggcctaggcttttgcaaaaagcttgattcttct 4070
gacacaacagtctcgaacttaaggctagagccaccatgattgaacaagatggatt 4125
gcacgcaggttctccggccgcttgggtggagaggctattcggctatgactgggca 4180
caacagacaatcggctgctctgatgccgccgtgttccggctgtcagcgcaggggc 4235
gcccggttctttttgtcaagaccgacctgtccggtgccctgaatgaactgcagga 4290
cgaggcagcgcggctatcgtggctggccacgacgggcgttccttgcgcagctgtg 4345
ctcgacgttgtcactgaagcgggaagggactggctgctattgggcgaagtgccgg 4400
ggcaggatctcctgtcatctcaccttgctcctgccgagaaagtatccatcatggc 4455
tgatgcaatgcggcggctgcatacgcttgatccggctacctgcccattcgaccac 4510
caagcgaaacatcgcatcgagcgagcacgtactcggatggaagccggtcttgtcg 4565
atcaggatgatctggacgaagagcatcaggggctcgcgccagccgaactgttcgc 4620
caggctcaaggcgcgcatgcccgacggcgaggatctcgtcgtgacccatggcgat 4675
gcctgcttgccgaatatcatggtggaaaatggccgcttttctggattcatcgact 4730
gtggccggctgggtgtggcggaccgctatcaggacatagcgttggctacccgtga 4785
tattgctgaagagcttggcggcgaatgggctgaccgcttcctcgtgctttacggt 4840
atcgccgctcccgattcgcagcgcatcgccttctatcgccttcttgacgagttct 4895
tctgagcgggactctggggttcgaaatgaccgaccaagcgacgcccaacctgcca 4950
tcacgatggccgcaataaaatatctttattttcattacatctgtgtgttggtttt 5005
ttgtgtgaatcgatagcgataaggatccgcgtatggtgcactctcagtacaatct 5060
gctctgatgccgcatagttaagccagccccgacacccgccaacacccgctgacgc 5115
gccctgacgggcttgtctgctcccggcatccgcttacagacaagctgtgaccgtc 5170
tccgggagctgcatgtgtcagaggttttcaccgtcatcaccgaaacgcgcgagac 5225
gaaagggcctcgtgatacgcctatttttataggttaatgtcatgataataatggt 5280
ttcttagacgtcaggtggcacttttcggggaaatgtgcgcggaacccctatttgt 5335
ttatttttctaaatacattcaaatatgtatccgctcatgagacaataaccctgat 5390
aaatgcttcaataatattgaaaaaggaagagtatgagtattcaacatttccgtgt 5445
cgcccttattcccttttttgcggcattttgccttcctgtttttgctcacccagaa 5500
acgctggtgaaagtaaaagatgctgaagatcagttgggtgcacgagtgggttaca 5555
tcgaactggatctcaacagcggtaagatccttgagagttttcgccccgaagaacg 5610
ttttccaatgatgagcacttttaaagttctgctatgtggcgcggtattatcccgt 5665
attgacgccgggcaagagcaactcggtcgccgcatacactattctcagaatgact 5720
tggttgagtactcaccagtcacagaaaagcatcttacggatggcatgacagtaag 5775
agaattatgcagtgctgccataaccatgagtgataacactgcggccaacttactt 5830
ctgacaacgatcggaggaccgaaggagctaaccgcttttttgcacaacatggggg 5885
atcatgtaactcgccttgatcgttgggaaccggagctgaatgaagccataccaaa 5940
cgacgagcgtgacaccacgatgcctgtagcaatggcaacaacgttgcgcaaacta 5995
ttaactggcgaactacttactctagcttcccggcaacaattaatagactggatgg 6050
aggcggataaagttgcaggaccacttctgcgctcggcccttccggctggctggtt 6105
tattgctgataaatctggagccggtgagcgtgggtctcgcggtatcattgcagca 6160
ctggggccagatggtaagccctcccgtatcgtagttatctacacgacggggagtc 6215
aggcaactatggatgaacgaaatagacagatcgctgagataggtgcctcactgat 6270
taagcattggtaactgtcagaccaagtttactcatatatactttagattgattta 6325
aaacttcatttttaatttaaaaggatctaggtgaagatcctttttgataatctca 6380
tgaccaaaatcccttaacgtgagttttcgttccactgagcgtcagaccccgtaga 6435
aaagatcaaaggatcttcttgagatcctttttttctgcgcgtaatctgctgcttg 6490
caaacaaaaaaaccaccgctaccagcggtggtttgtttgccggatcaagagctac 6545
caactctttttccgaaggtaactggcttcagcagagcgcagataccaaatactgt 6600

```
tcttctagtgtagccgtagttaggccaccacttcaagaactctgtagcaccgcct     6655
acatacctcgctctgctaatcctgttaccagtggctgctgccagtggcgataagt     6710
cgtgtcttaccgggttggactcaagacgatagttaccggataaggcgcagcggtc     6765
gggctgaacggggggttcgtgcacacagcccagcttggagcgaacgacctacacc     6820
gaactgagatacctacagcgtgagctatgagaaagcgccacgcttcccgaaggga     6875
gaaaggcggacaggtatccggtaagcggcagggtcggaacaggagagcgcacgag     6930
ggagcttccagggggaaacgcctggtatctttatagtcctgtcgggtttcgccac     6985
ctctgacttgagcgtcgatttttgtgatgctcgtcaggggggcggagcctatgga     7040
aaaacgccagcaacgcggcctttttacggttcctggccttttgctggccttttgc     7095
tcacatggctcgacagatct ••• 7115
```

DNA Type: Synthetic DNA

Description: Rattus norvegicus GABA-A gamma2 long isoform mRNA (Gabrg2)

Created: Mar 31, 2014
Last Modified: Nov 15, 2024

Accession Number: AY574252.1
Code Number:

Sequence Author: Dr. Marcel Goldschen-Ohm

Comments: pUNIV vector suitable for mammalian cells and Xenopus laevis oocytes.

References:

1. Venkatachalan SP, Bushman JD, Mercado JL, Sancar F, Christopherson KR, Boileau AJ.
Optimized expression vector for ion channel studies in Xenopus oocytes and mammalian cells using alfalfa mosaic virus.
Pflugers Arch 2007 Apr;454:155-63
PubMed ID: 17146677

Embedded Files:
